# Supplementary material for: Black-hole-inspired thermal trapping with graded heat-conduction metadevices
Source: Natl Sci Rev. 2022 Aug 16;10(2):nwac159. doi: 10.1093/nsr/nwac159 (PMC10016200; doi:10.1093/nsr/nwac159)
Supplement: nwac159_Supplemental_File [file nwac159_supplemental_file.pdf]

Supplementary Materials for "Black-hole-inspired thermal trapping with graded heat-conduction metadevices"

Liujun Xu[1,2,3,#], Jinrong Liu[1,#], Peng Jin[1], Guoqiang Xu[2], Jiaxin Li[4], Xiaoping Ouyang[5], Ying Li[6,7], Cheng-Wei Qiu[2,*] and Jiping Huang[1,*]

[1]*Department of Physics, State Key Laboratory of Surface Physics, and Key Laboratory of Micro and Nano Photonic Structures (MOE), Fudan University, Shanghai 200438, China*

[2]*Department of Electrical and Computer Engineering, National University of Singapore, Singapore 117583, Singapore*

[3]*Graduate School of China Academy of Engineering Physics, Beijing 100193, China*

[4]*School of Mechatronics Engineering, Harbin Institute of Technology, Harbin 150001, China*

[5]*School of Materials Science and Engineering, Xiangtan University, Xiangtan 411105, China*

[6]*Interdisciplinary Center for Quantum Information, State Key Laboratory of Modern Optical Instrumentation, ZJU-Hangzhou Global Scientific and Technological Innovation Center, Zhejiang University, Hangzhou 310027, China*

[7]*International Joint Innovation Center, Key Laboratory of Advanced Micro/Nano Electronic Devices and Smart Systems of Zhejiang, The Electromagnetics Academy of Zhejiang University, Zhejiang University, Haining 314400, China*

[#]*These authors contributed equally to this work.*

[*]*Corresponding authors. Emails: chengwei.qiu@nus.edu.sg; jphuang@fudan.edu.cn*

Part I: Comparison between the imitated and realistic advection

The imitated advection

We consider a steady case with forward heat transfer (Fig. S1a) by setting a high temperature of T_h at $x/L=0$ and a low temperature of T_c at $x/L=1$. The governing equation of heat transfer in a graded heat-conduction metadevice is

$$\rho(x)C(x)\frac{\partial T}{\partial t}+\frac{\partial}{\partial x}\left(-\kappa(x)\frac{\partial T}{\partial x}\right)=0, \tag{S1}$$

with parameters of $\kappa(x)=\kappa_0 e^{\alpha x}$ and $\rho(x)C(x)=\rho_0 C_0 e^{\alpha x}$. The substitution of a trial solution described by $T_f=A_f e^{\gamma x}+B_f$ into Eq. (S1) yields $\gamma=-\alpha$. Considering the boundary conditions of $T_f(x/L=0)=T_h$ and $T_f(x/L=1)=T_c$, we derive $A_f=(T_h-T_c)/(1-e^{-\alpha L})$ and $B_f=(T_c-T_h e^{-\alpha L})/(1-e^{-\alpha L})$. For a steady case with backward heat transfer, the corresponding temperature of T_b satisfies $T_b(x/L=0)=T_c$ and $T_b(x/L=1)=T_h$. Therefore, T_f and T_b can be summarized as

$$T_f=\frac{(T_h-T_c)e^{-\alpha x}}{1-\ e^{-\alpha L}}+\frac{T_c-T_h e^{-\alpha L}}{1-\ e^{-\alpha L}}, \tag{S2a}$$

$$T_b=\frac{(T_c-T_h)e^{-\alpha x}}{1-\ e^{-\alpha L}}+\frac{T_h-T_c e^{-\alpha L}}{1-\ e^{-\alpha L}}. \tag{S2b}$$

We calculate the temperature at $x/L=0.5$ from Eqs. (S2a) and (S2b). Due to $T_f(x/L=0.5)\neq T_b(x/L=0.5)$, the temperature distributions are asymmetric in opposite directions, resulting from the imitated advection.

We also consider a transient case with forward heat transfer (Fig. S1c) by applying a time-periodic temperature of $T_p=A_0 e^{-i\omega t}+T_0$ at $x/L=0$, where ω is the angular frequency. The imaginary unit is denoted by $i=\sqrt{-1}$, and the real part of T_p makes sense. The right boundary is set with the open condition. We discuss a wavelike temperature solution described by $T_f=A_0 e^{i(\beta_f x-\omega t)}+T_0$, where β_f is the forward wave number. Since heat conduction

features dissipation, β_f is a complex number whose imaginary part denotes the spatial decay rate. We substitute the wavelike solution into Eq. (S1) and derive $\mathrm{Re}(\beta_f) = \sqrt{2}\varepsilon/4$ and $\mathrm{Im}(\beta_f) = (8\alpha\omega + 2\sqrt{2}\alpha^2 D_0 \varepsilon + \sqrt{2} D_0 \varepsilon^3)/(16\omega)$ with $\varepsilon = \sqrt{-\alpha^2 + \sqrt{\alpha^4 + 16\omega^2/D_0^2}}$. The method also applies to a transient case with backward heat transfer, corresponding to a temperature profile of T_b. Therefore, T_f and T_b can be rewritten as

$$T_f = A_0 e^{i(\beta_f x - \omega t)} + T_0 = A_0 e^{-\mathrm{Im}(\beta_f) x} e^{i(\mathrm{Re}(\beta_f) x - \omega t)} + T_0, \tag{S3a}$$

$$T_b = A_0 e^{i(\beta_b \bar{x} - \omega t)} + T_0 = A_0 e^{-\mathrm{Im}(\beta_b) \bar{x}} e^{i(\mathrm{Re}(\beta_b) \bar{x} - \omega t)} + T_0, \tag{S3b}$$

with a definition of $\bar{x} = x - L$. The parameters of β_f and β_b satisfies

$$\beta_f = \frac{\sqrt{2}\varepsilon}{4} + i\frac{8\alpha\omega + 2\sqrt{2}\alpha^2 D_0 \varepsilon + \sqrt{2} D_0 \varepsilon^3}{16\omega}, \tag{S4a}$$

$$\beta_b = \frac{-\sqrt{2}\varepsilon}{4} + i\frac{8\alpha\omega - 2\sqrt{2}\alpha^2 D_0 \varepsilon - \sqrt{2} D_0 \varepsilon^3}{16\omega}. \tag{S4b}$$

Due to $|\mathrm{Im}(\beta_f)| \neq |\mathrm{Im}(\beta_b)|$ (i.e., different spatial decay rates), the temperature profiles are asymmetric in opposite directions, resulting from the imitated advection. Therefore, the imitated advection can lead to asymmetric heat transfer in steady and transient states.

The realistic advection

Heat transfer with diffusion and advection is described by

$$\frac{\partial T}{\partial t} + v_0 \frac{\partial T}{\partial x} - D_0 \frac{\partial^2 T}{\partial x^2} = 0, \tag{S5}$$

where v_0 is the advection velocity and $D_0 = \kappa_0/(\rho_0 C_0)$ is the thermal diffusivity. We discuss steady heat transfer and derive the forward and backward temperature distributions,

$$T_f = \frac{(T_h - T_c) e^{v_0 x/D_0}}{1 - e^{v_0 L/D_0}} + \frac{T_c - T_h e^{v_0 L/D_0}}{1 - e^{v_0 L/D_0}}, \tag{S6a}$$

$$T_b = \frac{(T_c - T_h) e^{v_0 x/D_0}}{1 - e^{v_0 L/D_0}} + \frac{T_h - T_c e^{v_0 L/D_0}}{1 - e^{v_0 L/D_0}}. \tag{S6b}$$

We further consider transient heat transfer, and the forward and backward wave numbers can be expressed as

$$\beta_f = \frac{\sqrt{2}\varepsilon}{4} + i\frac{-8v_0\omega + 2\sqrt{2}v_0^2\varepsilon + \sqrt{2}D_0^2\varepsilon^3}{16\omega D_0}, \tag{S7a}$$

$$\beta_b = \frac{-\sqrt{2}\varepsilon}{4} + i\frac{-8v_0\omega - 2\sqrt{2}v_0^2\varepsilon - \sqrt{2}D_0^2\varepsilon^3}{16\omega D_0}, \tag{S7b}$$

with a definition of $\varepsilon = \sqrt{-v_0^2/D_0^2 + \sqrt{v_0^4/D_0^4 + 16\omega^2/D_0^2}}$. Therefore, when the requirement of $v_0 = -\alpha D_0$ is satisfied, the imitated and realistic advection demonstrates a similar temperature field effect in steady and transient states.

To confirm these theoretical analyses, we perform finite-element simulations with two templates of COMSOL Multiphysics, i.e., heat transfer in solids and heat transfer in fluids. The requirement of $v_0 = -\alpha D_0$ is always satisfied. The steady results of the imitated advection are presented with solid lines, and those of the realistic advection are displayed with dotted lines (Fig. S1b). The transient results are shown in Fig. S1d. There is no temperature difference between the imitated and realistic advection. Meanwhile, the imitated advection can also lead to asymmetric heat transfer in steady and transient cases, just like the realistic advection. In other words, in terms of the temperature field effect, the imitated advection in graded heat-conduction metadevices is similar to the realistic advection induced by mass transfer. Consequently, graded heat-conduction metadevices can help reveal those advection-required phenomena and applications in completely stationary solids without external drives.

Part II: Thermal trapping in one dimension

Graded metadevices have broad applications for wave control. A typical example is focusing waves by a slab lens (Fig. S2a). The underlying mechanism is that the graded refractive index can produce an effective momentum. Similarly, since graded heat-conduction metadevices can generate the imitated advection, thermal trapping can also be expected to move hot spots flexibly and trap them elsewhere (Fig. S2b).

When a Gaussian wave packet is in the left part of the system (Fig. S2c1), we can design a graded heat-conduction metadevice with opposite imitated advection to trap the wave packet towards the central interface (Fig. S2c2 and c3). The quantitative data are also presented in Fig. S2d. We also plot the temperature evolution in a homogeneous material for reference (Fig. S2e). The hot spot is trapped in the graded heat-conduction metadevice but keeps almost stationary in the homogeneous material. Meanwhile, the temperature field amplitude decreases because heat conduction corresponds to dissipation. This design conveniently manipulates temperature profiles in completely stationary solids because energy-consuming external drives are no longer required. Such a one-dimensional case also lays a solid foundation for designing thermal trapping in high dimensions.

Part III: The imitated advection in high dimensions

Two dimensions

We consider a diffusion-advection process dominated by

$$\rho_0 C_0 \frac{\partial T}{\partial t} + \nabla \cdot (\rho_0 C_0 \boldsymbol{v}_0 T - \kappa_0 \nabla T) = 0. \tag{S8}$$

Mass conservation yields $\nabla \cdot (\rho_0 \boldsymbol{v}_0) = 0$ and further leads to $\nabla \cdot \boldsymbol{v}_0 = 0$ due to the constant value of ρ_0. Therefore, Eq. (S8) can be rewritten as

$$\rho_0 C_0 \frac{\partial T}{\partial t} + \rho_0 C_0 \boldsymbol{v}_0 \cdot \nabla T + \nabla \cdot (-\kappa_0 \nabla T) = 0. \tag{S9}$$

For two dimensions with cylindrical coordinates of (r, θ), Eq. (S9) can be reduced to

$$\frac{\partial T}{\partial t} + v_0 \frac{\partial T}{\partial r} - D_0 \left(\frac{1}{r} \frac{\partial}{\partial r} \left(r \frac{\partial T}{\partial r} \right) \right) = 0, \tag{S10}$$

where v_0 is along the radial direction and $\partial T / \partial \theta = 0$. The realistic advection term is $v_0 \partial T / \partial r$ in Eq. (S10). The imitated advection term also appears if we reduce Eq. (S10) to

$$\frac{\partial T}{\partial t} + v_0 \frac{\partial T}{\partial r} - \frac{D_0}{r} \frac{\partial T}{\partial r} - D_0 \frac{\partial^2 T}{\partial r^2} = 0, \tag{S11}$$

where $-D_0\partial T/(r\partial r)$ is the imitated advection term and $-D_0/r$ is the imitated advection velocity. However, $-D_0\partial T/(r\partial r)$ essentially results from the cylindrical coordinate system, which is not our interest. We aim to realize $v_0\partial T/\partial r$ with graded parameters. For this purpose, we consider the parameters have radial variations,

$$\kappa(r) = \kappa_0 e^{\alpha r}, \tag{S12a}$$

$$\rho(r)C(r) = \rho_0 C_0 e^{\alpha r}. \tag{S12b}$$

Then we substitute Eqs. (S12a) and (S12b) into the governing equation of heat conduction,

$$\rho(r)C(r)\frac{\partial T}{\partial t} + \frac{1}{r}\frac{\partial}{\partial r}\left(-r\kappa(r)\frac{\partial T}{\partial r}\right) = 0. \tag{S13}$$

We further reduce Eq. (S13) to

$$\frac{\partial T}{\partial t} + v_{i0}\frac{\partial T}{\partial r} - \frac{D_0}{r}\frac{\partial T}{\partial r} - D_0\frac{\partial^2 T}{\partial r^2} = 0, \tag{S14}$$

with a definition of $v_{i0} = -\alpha D_0$. Since Eqs. (S14) and (S11) share the same equation forms, the imitated advection can be achieved with graded parameters in two dimensions.

Three dimensions

We consider a three-dimensional case with spherical coordinates (r, θ, φ), and Eq. (S9) can be simplified as

$$\frac{\partial T}{\partial t} + v_0\frac{\partial T}{\partial r} - D_0\left(\frac{1}{r^2}\frac{\partial}{\partial r}\left(r^2\frac{\partial T}{\partial r}\right)\right) = 0, \tag{S15}$$

where v_0 is also along the radial direction and $\partial T/\partial\theta = \partial T/\partial\varphi = 0$. Similar to the above two dimensions, besides the realistic advection term of $v_0\partial T/\partial r$, the imitated advection term also appears if we simplify Eq. (S15) as

$$\frac{\partial T}{\partial t} + v_0\frac{\partial T}{\partial r} - \frac{2D_0}{r}\frac{\partial T}{\partial r} - D_0\frac{\partial^2 T}{\partial r^2} = 0, \tag{S16}$$

where $-2D_0\partial T/(r\partial r)$ is the imitated advection term. We also consider graded parameters with radial distributions,

$$\kappa(r) = \kappa_0 e^{\alpha r}, \tag{S17a}$$

$$\rho(r)C(r) = \rho_0 C_0 e^{\alpha r}. \tag{S17b}$$

With Eqs. (S17a) and (S17b), the governing equation of heat conduction becomes

$$\rho(r)C(r)\frac{\partial T}{\partial t} + \frac{1}{r^2}\frac{\partial}{\partial r}\left(-r^2\kappa(r)\frac{\partial T}{\partial r}\right) = 0. \tag{S18}$$

Then Eq. (S18) can be simplified as

$$\frac{\partial T}{\partial t} + v_{i0}\frac{\partial T}{\partial r} - \frac{2D_0}{r}\frac{\partial T}{\partial r} - D_0\frac{\partial^2 T}{\partial r^2} = 0, \tag{S19}$$

with a definition of $v_{i0} = -\alpha D_0$. Therefore, the imitated advection of our concern is also obtained in three dimensions.

Therefore, the exponential forms of thermal conductivity and the product of mass density and heat capacity work in one, two, and three dimensions and generate a constant imitated advection velocity of $v_{i0} = -\alpha D_0$. The exponential forms are not mandatory as long as $\partial\kappa(r)/\partial r \neq 0$ because the imitated advection results from graded thermal conductivities.

Part IV: Relation between graded parameters and curvilinear spacetime

The transformation theory [1-5] suggests that graded parameters are equivalent to curvilinear spacetime. Since the general transformation theory leads to anisotropic parameters [1,2] that are inconvenient for experimental implementation, we consider the conformal transformation theory [3-5]. Specifically, we discuss a virtual space of $w = m + \mathrm{i}n$ and a physical space of $z = x + \mathrm{i}y$, which are mapped by an analytical function of $w = f(z)$ and satisfy the Cauchy-Riemann conditions given by $\partial x/\partial m = \partial y/\partial n$ and $\partial x/\partial n = -\partial y/\partial m$. As the theory of conformal transformation optics suggests [3-5], the optical path in the w space is the same as that in the z space. The concept of optical path should be replaced by heat flux in thermotics. The heat flux in the w space is the same as in the z space. The heat flux

in the z space (denoted by J_z) is determined by

$$J_z = -\kappa_z \nabla_z T. \tag{S20}$$

Similarly, the heat flux in the w space (denoted by J_w) is described by

$$J_w = -\kappa_w \nabla_w T. \tag{S21}$$

The conformal transformation of $w = f(z)$ zooms in the z space with a ratio of

$$|f'(z)| = \left|\frac{dw}{dz}\right|. \tag{S22}$$

Therefore, the gradient operations in these two spaces satisfy

$$\nabla_z = \left|\frac{dw}{dz}\right| \nabla_w. \tag{S23}$$

To ensure the same heat fluxes (i.e., $J_z = J_w$), we can obtain

$$\kappa_z \nabla_z T = \kappa_z \left|\frac{dw}{dz}\right| \nabla_w T = \kappa_w \nabla_w T. \tag{S24}$$

Then we can derive the thermal conductivity transformation,

$$\kappa_z = \kappa_w \left|\frac{dw}{dz}\right|^{-1}. \tag{S25}$$

To discuss the transformation principle of the product of mass density and heat capacity, we further consider the governing equation of heat diffusion in the z space or that in the w space,

$$\rho_z C_z \frac{\partial T}{\partial t} - \kappa_z \nabla_z^2 T = 0, \tag{S26}$$

$$\rho_w C_w \frac{\partial T}{\partial t} - \kappa_w \nabla_w^2 T = 0. \tag{S27}$$

The substitution of Eqs. (S23) and (S25) into Eq. (S26) yields

$$\rho_z C_z \frac{\partial T}{\partial t} - \kappa_w \left|\frac{dw}{dz}\right|^{-1} \left|\frac{dw}{dz}\right|^{2} \nabla_w^2 T = 0. \tag{S28}$$

Comparing Eqs. (S27) and (S28), we derive

$$\rho_z C_z = \rho_w C_w \left|\frac{dw}{dz}\right|. \tag{S29}$$

Therefore, the theory of conformal transformation thermotics can be summarized as

$$\kappa_z = \kappa_w \left|\frac{dw}{dz}\right|^{-1}, \tag{S30a}$$

$$\rho_z C_z = \rho_w C_w \left|\frac{dw}{dz}\right|. \tag{S30b}$$

We consider the following conformal transformation, usually used to design the trapping effect in optics,

$$w = \ln z. \tag{S31}$$

Then we derive the graded thermal parameters from Eqs. (S30a) and (S30b),

$$\kappa_z = \kappa_w r, \tag{S32a}$$

$$\rho_z C_z = \rho_w C_w r^{-1}, \tag{S32b}$$

with a definition of $r = |z| = \sqrt{x^2 + y^2}$. The graded thermal parameters described by Eqs. (S32a) and (S32b) also lead to the imitated advection pointing towards the center. Therefore, the parameters applied in the main text satisfy the requirement by designing the imitated advection pointing towards the center.

To summarize, the theory of conformal transformation thermotics linking graded parameters and curvilinear spaces provides a qualitative explanation of the physical basis between our designed structures and thermal trapping.

References

[1] Pendry JB, Schurig D and Smith DR. Controlling electromagnetic fields. *Science* 2006, **312**: 1780-2.

[2] Leonhardt U and Philbin TG. General relativity in electrical engineering. *New J Phys* 2006; **8**: 247.

[3] Leonhardt U. Optical conformal mapping. *Science* 2006; **312**: 1777-80.

[4] Xu L and Chen H. Conformal transformation optics. *Nat. Photonics* 2015; **9**: 15-23.

[5] Ohno H and Usui T. Gradient-index dark hole based on conformal mapping with etendue conservation. *Opt Express* 2019; **27**: 18493-507.

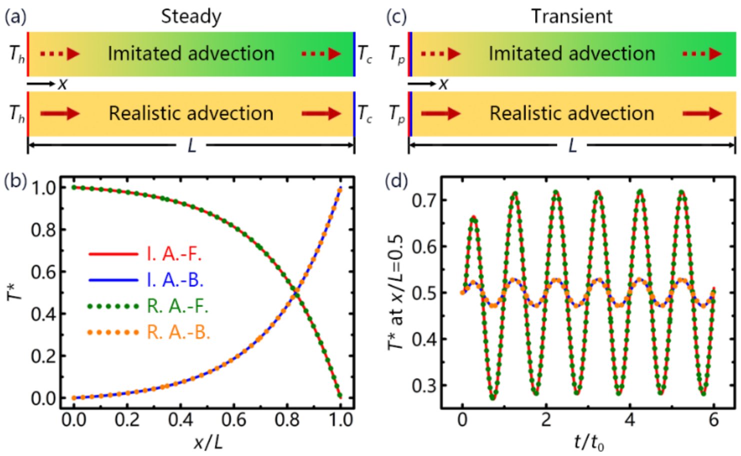

Figure S1. Comparison between the imitated advection (I. A.) and realistic advection (R. A.). (a) and (b) Steady case with T^* as a function of x/L. (c) and (d) Transient case with T^* as a function of t/t_0 at $x/L = 0.5$. $L = 0.2$ m, $\kappa_0 = 400$ W m^{-1} K^{-1}, $\rho_0 = 8900$ kg/m^3, $C_0 = 390$ J kg^{-1} K^{-1}, $\alpha = -20$ m^{-1}, $T_p = 0.5(T_h - T_c)\cos(2\pi t/t_0) + 0.5(T_h + T_c)$ K, $T_h = 363$ K, $T_c = 283$ K, and $t_0 = 100$ s. F.: Forward; B.: Backward.

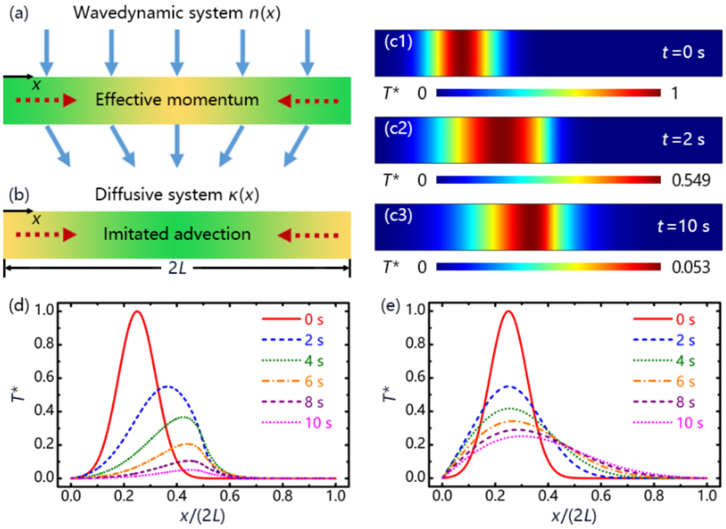

Figure S2. One-dimensional thermal trapping. (a) Wave focusing by a graded refractive index. (b) Thermal trapping by a graded thermal conductivity. (c1)-(c3) Simulated results of thermal trapping at 0, 2, and 10 s, respectively. (d) T^* as a function of $x/(2L)$. (e) T^* as a function of $x/(2L)$ in a homogeneous medium with constant thermal diffusivity of D_0. $L = 0.1$ m, $\kappa_0 = 400$ W m^{-1} K^{-1}, $\rho_0 = 8900$ kg/m^3, $C_0 = 390$ J kg^{-1} K^{-1}, and $\alpha = -100$ m^{-1}. The initial normalized temperature is set at $T^* = e^{-(50x-2.5)^2}$, and the normalized temperatures at the left and right boundaries are set at $T^* = 0$.
